# Optimized LightGBM Power Fingerprint Identification Based on Entropy Features

**DOI:** 10.3390/e24111558

**Published:** 2022-10-29

**Authors:** Lin Lin, Jie Zhang, Na Zhang, Jiancheng Shi, Cheng Chen

**Affiliations:** 1 College of Information and Control Engineering, Jilin Institute of Chemical Technology, Jilin 132022, China; 2 State Grid Liaoning Economic Research Institute, Shenyang 110015, China

**Keywords:** power fingerprint, entropy feature, Boruta algorithm, Optuna algorithm, LightGBM

## Abstract

The huge amount of power fingerprint data often has the problem of unbalanced categories and is difficult to upload by the limited data transmission rate for IoT communications. An optimized LightGBM power fingerprint extraction and identification method based on entropy features is proposed. First, the voltage and current signals were extracted on the basis of the time-domain features and V-I trajectory features, and a 56-dimensional original feature set containing six entropy features was constructed. Then, the Boruta algorithm with a light gradient boosting machine (LightGBM) as the base learner was used for feature selection of the original feature set, and a 23-dimensional optimal feature subset containing five entropy features was determined. Finally, the Optuna algorithm was used to optimize the hyperparameters of the LightGBM classifier. The classification performance of the power fingerprint identification model on imbalanced datasets was further improved by improving the loss function of the LightGBM model. The experimental results prove that the method can effectively reduce the computational complexity of feature extraction and reduce the amount of power fingerprint data transmission. It meets the recognition accuracy and efficiency requirements of a massive power fingerprint identification system.

## 1. Introduction

Traditional non-intrusive load disaggregation (NILD) requires the measurement of relevant electrical quantities from the power inlet and the processing and analysis of signals such as voltages and currents [1]. The different electrical devices operate with different electrical quantities, presenting a unique power fingerprint of each electrical device. The identification of customers’ appliances through the power fingerprint features has become a research hotspot in the field of load monitoring because of its operability, low implementation cost, and high customer acceptance [2]. However, as the power fingerprint data in the distribution network becomes increasingly larger, the transmission of massive power fingerprint data puts huge pressure on the bandwidth of the communication network.

IoT communication technology provides a new approach to building a reliable power fingerprint monitoring system. A large amount of power fingerprint data can be obtained through low-cost edge-side collection devices [3]. The authors of [4] investigated a load-monitoring approach based on collaborative computing between edge devices and edge data centers. Reference [5] proposed an edge-computing architecture for load identification in home scenarios that can significantly reduce the amount of data transmission over the network. Due to the data transmission rate limitation of NB-IoT, LoRa, and other IoT communication technologies, it is difficult to upload the raw signal directly to the upper layer system for power fingerprint identification. Therefore, the original signal should be feature extracted at the edge side, and then the valid power fingerprint feature data should be uploaded to reduce the amount of system data transmission.

In NILD, the power fingerprint process often contains two key aspects, feature extraction and load identification. Earlier, the steady state characteristics of active power P and reactive power Q were often used for identification [6]. References [7,8] added features such as current waveform, harmonics, transient power waveform, and switching transient waveform for load identification based on the use of active and reactive power. In [9], the voltage and current signals were converted into two-dimensional images in combination with the V-I trajectory for load identification. In [10,11], the short-time Fourier transform (STFT) and wavelet transforms (WT) were used to transform the data in the time-frequency domain to extract the frequency domain features. The frequency domain features were then combined with other time domain features for load identification. Reference [12] proposed a systematic feature selection method to remove irrelevant features. An optimal subset of features for NILD was constructed and identified using a random forest algorithm. Most of the existing research on power fingerprint identification considers the extraction of load features such as the time domain and frequency domain, but there has not been any extraction and application of entropy features. The entropy feature is used to describe the uncertainty degree and complexity of the system. A higher entropy value indicates the higher complexity and disorder of the system [13]. Approximate entropy and sample entropy are both methods to measure the complexity of time series. Sample entropy is an improvement of the approximate entropy algorithm and a more widely used method to calculate the entropy characteristic value at present. The smaller the sample entropy of time series, the smaller its complexity and the higher its self-similarity [14]. Extracting time-frequency features from raw signals with high sampling rates can lead to high stress on data storage devices and data communication devices. Therefore, entropy features and other time-domain features are extracted from the original signal and combined with V-I trajectory features for load identification, thus reducing the computational complexity of feature extraction and the amount of data transmission.

Unoptimized raw features can reduce recognition efficiency and accuracy. Feature selection can be used to reduce feature dimensionality and improve recognition efficiency. The traditional recursive elimination method (RFE) [15] usually relies on a subset of features in the feature selection process, thus generating errors and losing some relevant features in the feature selection process. The Boruta algorithm [16] is a fully encapsulated feature selection method based on random forest (RF) that tries to capture all important features in the dataset associated with the outcome variable. However, using the random forest as the base learner is less efficient in finding the best features, so there is still room for improvement in training efficiency.

Machine learning is often applied to the study of power fingerprint identification. Shallow learning machine learning methods such as decision trees (DTs), support vector machines (SVMs), K-nearest neighbors (KNNs), and random forests [17,18,19] can be applied to power fingerprint identification with certain results. However, the accuracy of such machine learning classification methods can still be further improved. In [20], a convolutional neural network (CNN) in deep learning was used to identify power devices in different states after feature extraction. In [21], the recurrent neural network (RNN) was trained using time series data to successfully predict the power consumption of each power device. Deep-learning-based classifiers require high hardware configurations and long training times. It is difficult to meet the economic and real-time requirements. LightGBM is an integrated learning framework for boosting decision trees as weak classifiers [22]. Compared with CNN, gradient boosting decision tree (GBDT) [23], extreme gradient boosting (XGBoost) [24], and other algorithms, LightGBM has better accuracy and higher recognition efficiency [25]. On the one hand, compared with other machine learning models, LightGBM speeds up the training speed of GBDT models without reducing the accuracy, has stable recognition effects, and reduces the training time. On the other hand, compared with common deep learning models such as CNN, LightGBM has relatively simple structural parameters and requires fewer optimization parameters. Due to the category imbalance problem of massive power fingerprint data, the classification performance of LightGBM for minority sample categories will be degraded if the weights of minority sample categories are not considered. Commonly used balancing data methods are broadly classified into data-based and model-based methods [26]. Data-based methods such as that of the reference [27] use SMOTE to extend the NILD dataset by a small number of samples so that the number of samples is equal for all classes. Conversely, model-based methods involve reweighting the loss function or directly modifying the loss function [28]. In addition, the classification performance and efficiency of LightGBM are closely related to the hyperparameter values of the model. Reference [29] used a simple brute force method Grid Search to optimize the parameters, but the cost of the brute force search is high. Reference [30] used a random search and Bayesian parameter optimization method to avoid many redundant operations performed by Grid Search, but there was randomness and volatility in its optimization search process. The default hyperparameter settings as well as the above-mentioned methods are difficult in terms of achieving the best classification performance.

In this paper, a new method of power fingerprint extraction and identification method based on entropy features is proposed. First, time domain features and V-I trajectory features were extracted from the voltage and current signals of electrical equipment to construct a 56-dimensional original feature set containing six entropy features. Then, the Boruta algorithm with LightGBM as the base learner was used for feature selection of candidate features to determine the 23-dimensional optimal feature subset containing five entropy features. After that, the optimal feature subset was calculated at the edge side and uploaded to the upper system for analysis. Finally, the loss function of LightGBM was improved and the weights for a few sample categories were increased in the training. A classifier based on the Optuna optimized parameter algorithm of LightGBM was constructed in the upper system for the power fingerprint. The COOLL public dataset [31] was used for experiments to verify the effectiveness and advancement of the method.

## 2. The Power Fingerprint Identification Architecture

Edge-side power fingerprinting devices are usually installed on the residential load side to collect data. Considering that all the data must be uploaded to the upper layer system, it will generate a large communication pressure and cost. Thus, for the raw signal to extract the relevant features through edge-side devices instead of uploading the raw signal directly, this can effectively reduce the communication pressure and cost of the system.

**Figure 1:**
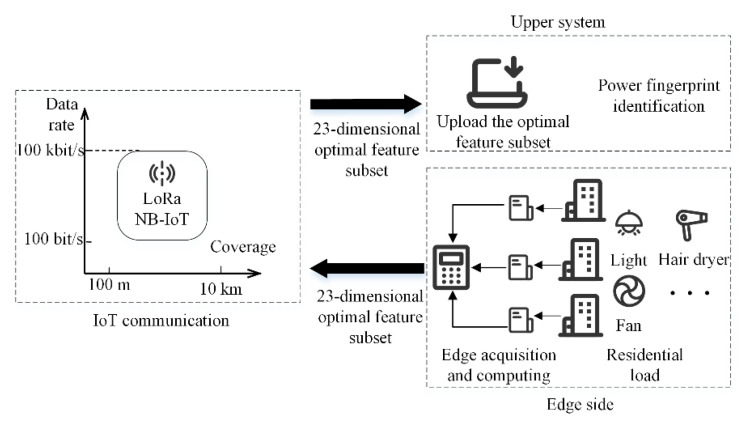
The power fingerprint identification architecture based on IoT communication.

The use of NB-IoT, LoRa, and other IoT communication methods for communication needs to consider the impact of a limited data transmission rate. For example, the coverage range of IoT communication methods NB-IoT and LoRa is 10 km, and the maximum data transmission rate is 100 kbit/s [32]. To meet the narrow-width IoT data transmission rate constraint, Figure 1 illustrates the application of the narrow-width IoT communication method to the power fingerprint identification architecture in this paper.

To ensure the effective application of IoT technology, the system needs to be designed to consider the data transmission rate limitation of this communication method. First, edge acquisition and feature calculation devices are installed at the edge side. The voltage and current signals of residential domestic loads are acquired, and the optimal subset of features that have been determined are calculated. Then, the optimal set of features are uploaded to the upper system using narrow-width IoT communication. Finally, the power fingerprint identification is performed in the upper system. This paper focuses on the edge feature extraction and power fingerprint identification algorithm that satisfies the narrow-width IoT communication method of the above architecture. The system architecture is presented only as background for the analysis in this paper.

## 3. Feature Extraction Based on Time-Domain Analysis and V-I Trajectory

**Figure 2:**
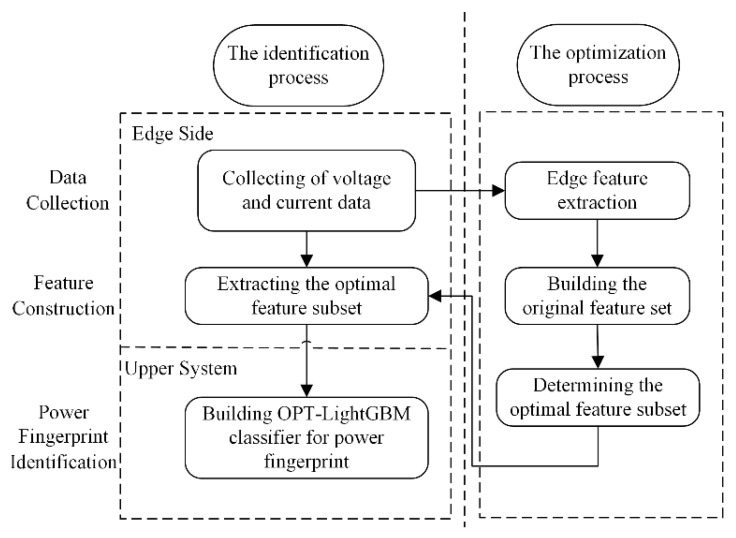
The power fingerprint identification process.

Figure 2 is the basic process of the proposed power fingerprint identification method. The voltage and current data are collected for edge feature extraction to determine the optimal feature subset. The optimal feature subset is extracted instead of the original signal upload, which can meet the bandwidth constraint of the low-cost, low-communication, narrow-width IoT communication method. Therefore, the signal features can be extracted by the edge-side device instead of the original signal for the upper system analysis. In the upper system, a classifier is constructed to perform power fingerprinting on the optimal feature subset.

Edge computing devices have limited computing power and are limited by the IoT transmission bandwidth. Without losing identification accuracy, higher demands are placed on the amount of feature extraction computation and the amount of data uploaded through the IoT by edge-side devices. Therefore, the design of related algorithms needs to fully consider the edge-side computational pressure to reduce the complexity of feature extraction methods and reduce the hardware cost of edge-side devices. First, the new method extracts 24 features on the basis of time-domain features for high-frequency current signals [12]; in addition to the traditional time-domain features, six entropy features are included to better characterize the complexity and self-similarity of current signals in a steady state. Then, 32 V-I trajectory features are extracted by combining voltage and current trajectories in a steady state and transient state [33,34]. Finally, the above 56-dimensional features are used to construct the original feature set. In the experiments, the sampling rate of the original signal is 100 kHz. The duration of the original signal is 6 s. The number of cycles in the experimental sample is 1. This paper extracts the relevant features on the basis of the voltage and current signals in steady state and transient state within one cycle, respectively. 

Table 1 and Table 2 list the calculation formulas and feature numbers of current features and entropy features, where x(n)=1,2,⋯,N is the amplitude corresponding to the nth sampling point, N is the total number of sampling points, $p_{n}$ is the probability density of the nth sampling point, and α is the parameter for entropy calculation. Construct the time series x(n) as an m-dimensional vector, $x_{m}(n)=\left\{x(n),x(n+1),\cdots ,x(n+m-1)\right\},n=1,2,\cdots N-m+1$. Define $d_{nj}^{m}$ to be the distance between vectors $x_{m}(n)$ and $x_{m}(j)$ as $d_{nj}^{m}=\max(\left|x(n+k)-x(j-k)\right|),k=0,1,\cdots m$. Define $C_{n}^{m}(r)$ as the probability that the distance between any vector $x_{m}(n)$ and $x_{m}(j)$ is less than r, $C_{n}^{m}(r)=\frac{\sum_{j=1}^{N-m+1}\theta (d_{nj}^{m}-r)}{N-m+1}$, where θ is the Heaviside function [35]. $\Phi^{m}(r)={(N-m+1)}^{-1}\sum_{n=1}^{N-m+1}\ln C_{n}^{m}(r)$, $\Psi^{m}(r)={(N-m+1)}^{-1}\sum_{n=1}^{N-m+1}C_{n}^{m}(r)$, $B^{m}(r)={\left(N-m\right)}^{-1}\sum_{n=1}^{N-m}C_{n}^{m}(r)$, where m is the embedding dimension, and r is the similarity tolerance. Table 3 and Table 4 list the feature categories and numbers of 32 V-I trajectory features, respectively; the calculation formulas are shown Table A1 and Table A2 in Appendix A.

**Figure 3:**
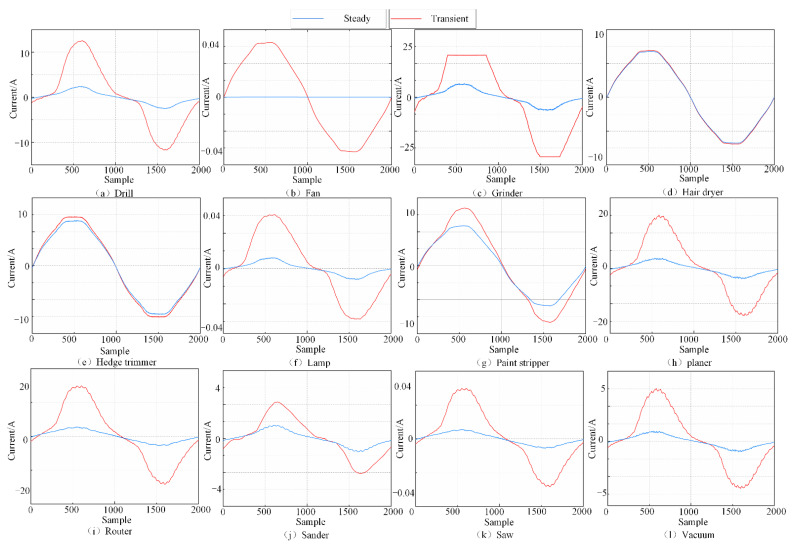
Voltage and current signal diagram. (**a**–**l**) show the voltage and current waveforms of 12 types of electrical equipment in the COOLL dataset in different states.

The experiments were performed on a microcomputer configured with an AMD R7-5700 CPU and DDR4 3200 MHz 16 GB memory. The experimental subjects were all conducted under the COOLL public dataset with a raw signal duration of 6 s and a sampling frequency of 100 kHz. Figure 3 is the current signals of 12 electrical devices in the COOLL dataset in transient and steady states given in one cycle. It can be seen in Figure 3 that the current signals of different electrical devices in the steady state and transient operation were different. By analyzing the time domain waveforms of the current signals, the current signal characteristics of different electrical devices can be extracted.

**Figure 4:**
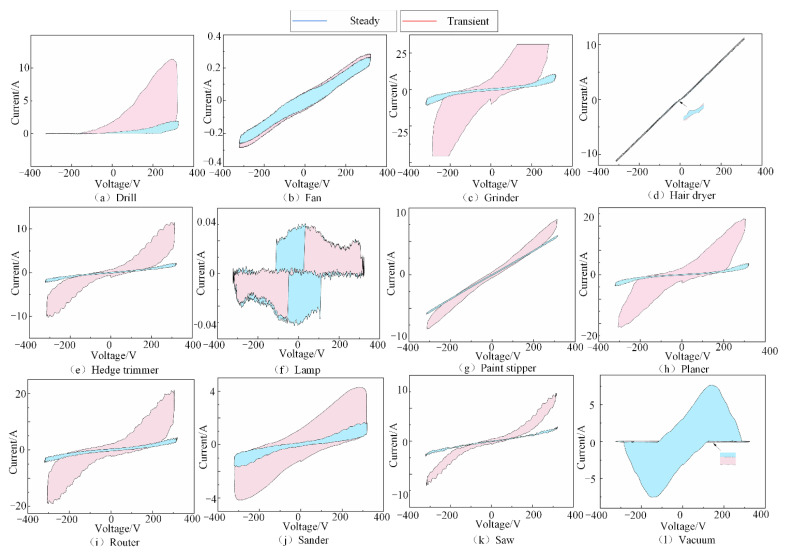
V-I trajectory images. (**a**–**l**) show the V-I trajectory images of 12 types of electrical equipment in the COOLL dataset in different states.

Figure 4 shows the V-I trajectory images of 12 types of electrical equipment in transient and steady states. As can be seen from Figure 4, the V-I trajectory images had significant differences for different types of electrical loads. The V-I trajectory feature of different electrical devices can be extracted by analyzing the shape information of the V-I trajectories.

## 4. Feature Selection Based on the Modified Boruta Algorithm

### 4.1. Boruta Algorithm

The Boruta algorithm is a feature selection algorithm based on a random forest base learner. It considers the fluctuations in the average accuracy loss of the tree in the random forest and uses it to measure the importance of the features [36]. The main idea of the Boruta algorithm is by evaluating the importance of each feature variable and then keeping the set of features marked as important.

**Figure 5:**
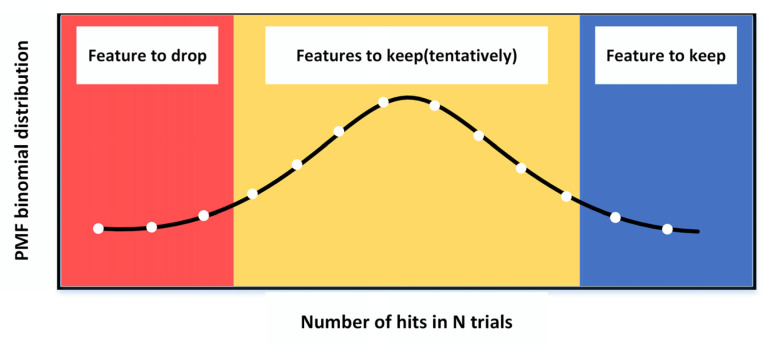
Binomial distribution of *n*-experiments.

The Boruta algorithm calculates the binomial distribution of feature hits by multiple iterations of feature selections, as shown in Figure 5. The red region is the rejection region, where features classified into this region are considered to be interference noise and are therefore removed directly. The yellow region is the hesitation region, where features classified to this region are somewhat predictable and need to be retained at their discretion. The blue region is the acceptance region, where features classified in this region are retained [36].

### 4.2. Modified Boruta Algorithm

To improve the optimization efficiency of the Boruta algorithm, LightGBM was used as the base learner to replace the random forest base learner on the basis of the Boruta algorithm. The specific algorithm steps are as follows:

Input: The original feature matrix $R=\left(r_{1},r_{2},\ldots ,r_{j}\right)$; the base learner M(θ) is LightGBM.

Step 1: For each original feature R, randomly disrupt the order. Duplicate the original features to obtain the shadow feature matrix S, and the new feature matrix N=[R,S] is formed by splicing with the original feature R.
Step 2: The new feature matrix N is used as input and the base learner M(θ) is trained. Calculate the importance score $Z_{\mathrm{score}\;}$.
  (1) $$Z_{\mathrm{score}\;}=\frac{E_{f}}{f}$$
  where $E_{f}$ is the evaluated importance of the feature, and f is the importance of the feature.
Step 3: Find the maximum value of the importance score in the shadow features and mark it as S_max. Mark the features with importance scores higher than S_max in the original features as important.
Step 4: Marking features with importance scores lower than S_max in the original feature as unimportant and permanently deleting them.
Step 5: Remove all shadow features and repeat the above process until all important features are filtered out.

Output: The optimal feature subset $S_{\max}$.

## 5. Construction of Power Fingerprint Identification Classifier

LightGBM is a new implementation of GBDT with faster training speed, shorter training time, and higher accuracy, which is widely used in classification tasks [22]. Its implementation is as follows:

The goal of each iteration round is to find the weak learner $f_{t}(x)$ such that the loss function of this round is minimized, i.e.,

(2) $$L\left(y,F_{t}(x)\right)=L\left(y,F_{t-1}(x)+f_{t}(x)\right)$$

where $F_{t-1}(x)$ represents the learner obtained in the previous iteration, and L() represents the loss function.

The negative gradient according to Formula (2) was used to obtain an approximation of the loss function for this round, i.e.,

(3) $$l_{t}=-\frac{\partial L\left(y_{i},F_{t-1}(x_{i})\right)}{\partial F_{t-1}(x_{i})}$$

The objective function is usually quadratic in variance, and $f_{t}(x)$ can be approximated as

(4) $$f_{t}(x)=\underset{f\in F}{\arg\min}\sum {\left(l_{t}-f_{t}(x)\right)}^{2}$$

The strong learner that obtains this iteration is

(5) $$F_{t}(x)=F_{t-1}(x)+f_{t}(x)$$

### 5.1. Improved LightGBM Model

The power fingerprint data in the real environment is characterized by class imbalance. The traditional classifier does not consider the class imbalance problem, which results in the classifier’s insufficient ability to recognize the minority sample class. To solve the above problem, the L2 regular term is first introduced to adjust the loss function of LightGBM, and then a higher weight is assigned to a few sample categories. When LightGBM samples data using the one-sided gradient sampling algorithm, it is easier to select data from the minority sample category to enhance the recognition performance of the LightGBM model on the imbalanced dataset. The specific improvements are as follows:

The improved loss function for the *t*th tree is

(6) $$L=-\frac{1}{N}(\sum_{i=1}^{N}\alpha_{i}L(y_{i},F_{t-1}(x_{i}))+\frac{\lambda }{2}{∥a∥}_{2}^{2})$$

(7) $$\alpha_{i}=\left\{ \begin{array}{l} 1,y_{i}=0\\ c,y_{i}=1 \end{array}\right.$$

where $\alpha_{i}$ is the coefficient of category weights; $L(y_{i},F_{t-1}(x_{i}))$ is the original loss function; λ is the regularization coefficient; and $y_{i}=0$ and $y_{i}=1$ represent normal sample labels and minority sample labels, respectively. The value of c is related to the sample proportion.

### 5.2. Optuna Optimization Algorithm

Machine learning models that want to improve their results require proper hyperparameter tuning. Hyperparameter optimization affects the output of a machine learning model. Hyperparameter optimization is a key process in machine learning for optimizing hyperparameters. Optuna is a framework for automated hyperparametric optimization that obtains the optimal solution by iteratively invoking and evaluating the objective function for different parameter values [37]. The specific features are as follows:

- *Define-by-run* framework: Optuna describes hyperparametric optimization as the process of maximizing or minimizing an objective function given a set of hyperparameters and returning its (validated) score [37]. The function does not depend on externally defined static variables and dynamically constructs the search space of the neural network structure (number of layers and number of hidden units).
- Efficient sampling: Optuna has both relational and independent sampling [38] and can identify trial results. These results provide information for concurrent relationships. The framework can identify potential co-occurrence relationships after a certain number of independent samples and use the inferred co-occurrence relationships for a user-selected relational sampling algorithm.
- Efficient pruning: Optuna periodically monitors intermediate target values and terminates trials that do not meet predefined conditions. It also uses the asynchronous successive halving algorithm [37], and therefore we can perform parallel computations here without much influence on each other.

### 5.3. Construction of the Optuna–LightGBM Classification Model

LightGBM has difficulty in determining some of the hyperparameter values due to the problem of there being many hyperparameters [30]. Therefore, the Optuna algorithm was used to optimize the hyperparameters of LightGBM. Following this, the model was trained using the adjusted parameters. The number of Optuna iterations was set to 50. The specific steps to construct the OPT–LightGBM optimization model are as follows:

1. Initialization, and then determining the direction of optimization, the type of parameters, the range of values, and the maximum number of iterations.
2. Enter the loop: selecting a set of individuals uniformly within the function defining the range of parameter values, automatically terminating hopeless individuals using a pruner according to the pruning conditions, and determining the value of the objective function for the overall number of uncomputed individuals.
3. Repeating the above steps for the loop and jumping out of the loop when the maximum number of iterations is reached.
4. Obtaining the best parameter values and the best values of the objective function and output the final model OPT–LightGBM.

**Figure 6:**
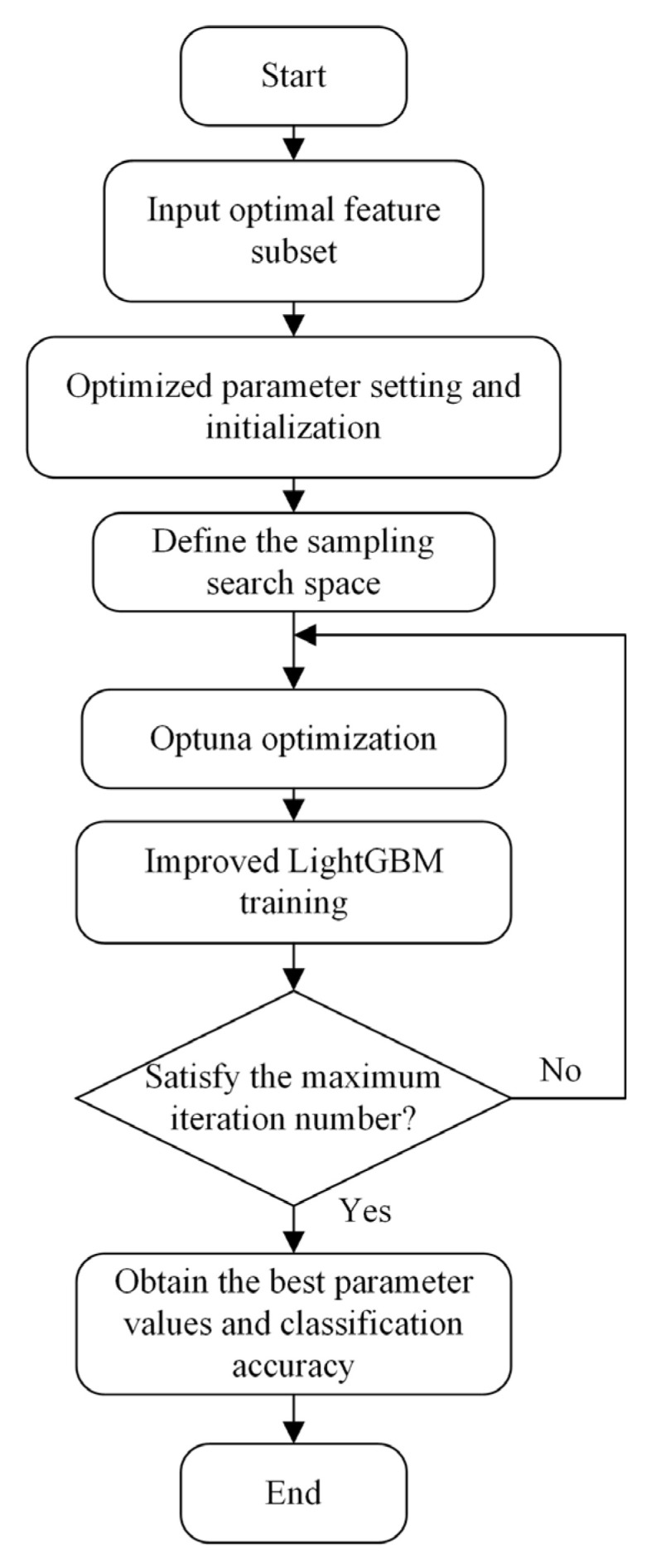
The specific process for the construction of the Optuna–LightGBM.

The specific process for the construction of the Optuna–LightGBM is shown in Figure 6. First, the optimal feature subset was input, the optimization parameters were set and initialized, and the search space was defined for adoption. Then, the parameters of the improved LightGBM were optimized and trained using Optuna after entering the loop. Finally, the best parameter values and classification accuracy were obtained when the maximum number of iterations was satisfied. The final model OPT–LightGBM was output.

### 5.4. Evaluation Metrics

To evaluate the classification performance of the OPT–LightGBM model more comprehensively, the evaluation metrics selected in this paper included Recall (*R*_re_), Precision ($P_{\mathrm{re}}$), and F1-score ($F_{\mathrm{score}}$) [30].

(8) $$R_{\mathrm{re}}=\frac{T_{p}}{T_{p}+F_{N}}$$

(9) $$P_{\mathrm{re}}=\frac{T_{p}}{T_{p}+F_{p}}$$

(10) $$F_{\mathrm{score}\;}=\frac{2P_{\mathrm{re}}R_{\mathrm{re}}}{P_{\mathrm{re}}+R_{\mathrm{re}}}$$

where $T_{p}$ is the number of appliances of a certain type correctly predicted, $F_{p}$ is the number of appliances of other types predicted to be of a certain type, and $F_{N}$ is the number of appliances of a certain type incorrectly predicted to be of other types.

## 6. Result and Discussion

### 6.1. Dataset Selection

To verify the effectiveness of the proposed method in this paper, the COOLL public dataset [31] was selected to carry out the power fingerprint identification experiments. The sampling frequency was 100 kHz, and the signal duration was 6 s. Table 5 is the COOLL dataset data that included 12 types of electrical appliances, and each appliance type is represented by examples of different labels, brands, and models, with a total of 840 sets of waveform data. In the experiment, 70% of the data were randomly selected as the training set and 30% of the data were used as the test set.

### 6.2. Construction of Optimal Feature Subsets Based on the Modified Boruta Algorithm

**Figure 7:**
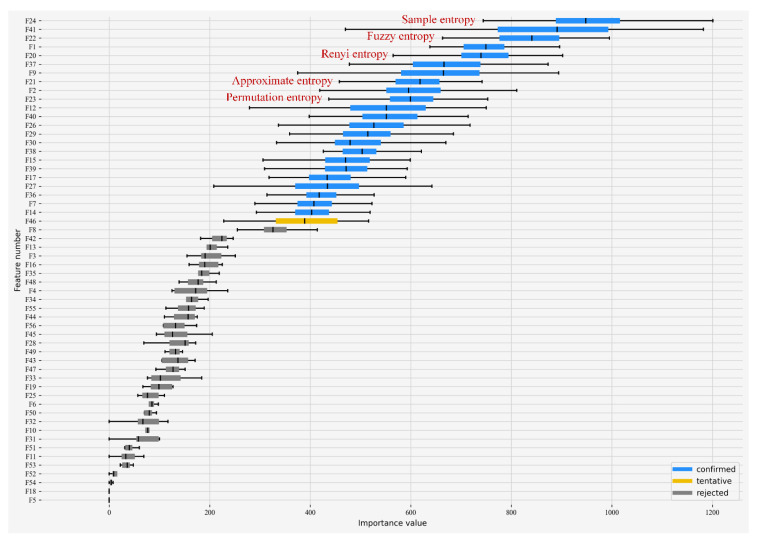
Feature importance ranking.

To further reduce the computational effort of edge-side feature extraction and to meet the demand of limited data transmission rate, the modified Boruta algorithm was used to perform feature selection on the original feature set. The number of iterations was 100, and the base model selected by the modified algorithm was LightGBM. Figure 7 shows that the Boruta algorithm based on LightGBM marked 23 features as important, 32 features as unimportant, and 1 feature (F46 feature) without giving a judgment after 100 iterations. The F46 feature that has not been judged for the time being belongs to the hesitant region, and it is necessary to decide whether to retain it. To reduce the computation demand of the edge side and the overall system construction and application cost as much as possible, the F46 feature was deleted in this paper. The Boruta algorithm formed the optimal feature subset from the features marked as important. The optimal feature subset after feature selection contains five entropy features, which were sample entropy, fuzzy entropy, Renyi entropy, approximate entropy, and permutation entropy, in that order.

**Figure 8:**
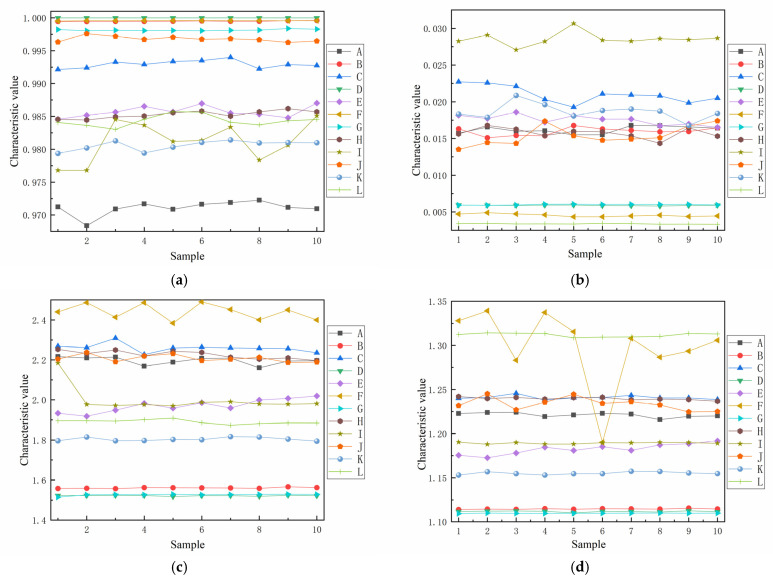
Distribution of different features. (**a**) Feature distribution of feature F24. (**b**) Feature distribution of feature F41. (**c**) Feature distribution of feature F7. (**d**) Feature distribution of feature F14.

To verify the validity of the importance measure of feature classification ability, the four features (F24, F41, F7, and F14) with the highest, higher, lower, and lowest importance values in the optimal feature subset were selected for comparison, where F24 denotes the sample entropy, F41 denotes the curvature of the mean line at steady state, F7 denotes the cliffness, and F14 denotes the waveform index. Figure 8 shows the feature distributions of the four features, where 10 groups of samples are taken for each type of appliance to demonstrate. It can be seen that when the sample entropy and the curvature of the mean line at steady-state features were used to describe the characteristics of different appliances, the degree of differentiation was high and the classification effect was good.

To verify the superiority of the modified Boruta algorithm, the method in this paper was compared with featureless selection, the correlation coefficient method, the recursive elimination method, the genetic algorithm (GA), and the embedded feature selection method (LightGBM) using LightGBM as the base classifier to carry out comparison experiments. The experimental results are shown in Table 6.

As can be seen from Table 6, the modified Boruta algorithm outperformed other feature selection algorithms in all metrics. Compared with featureless selection, they were 1.98%, 1.53%, 2.02%, and 1.93% higher in terms of accuracy, Recall, Precision, and F1-score, respectively. The experimental results verified that the method in this paper can effectively remove redundant features and reduce the model complexity without reducing the classification accuracy.

### 6.3. Amount of Data Transmission

To analyze the differences in the finite data transmission rate requirements of different methods, the amount of data required to upload the optimal feature subset, the original feature set, and the original signal were compared in this paper. The sampling rate was 100 kHz, and the single analysis data length was one cycle of voltage and current signal data. The amount of data required to upload a set of the optimal feature subset, the original feature set, and the original signal is shown in Table 7.

As can be seen from Table 7, when only the optimal feature subset was uploaded, the amount of data transmission was about 1118 bytes. The new method reduced the amount of data transmission requirement by 99.9% compared to uploading the original signal. The new method also reduced the amount of data transmission requirement by 29.7% compared to uploading the original feature set. Therefore, it can be seen that the new method effectively reduced the amount of data transmission required for power fingerprint data analysis. At the same time, the new method reduced the feature set dimension to 23 dimensions, and feature selection effectively reduced the amount of edge computation. It satisfies the basic data transmission requirements of the narrow-width IoT communication method.

### 6.4. Comparison of Different Hyperparameter Optimization Algorithms

After constructing the optimal feature subsets, the optimal parameters of the LightGBM model were obtained on the basis of the hyperparameter optimization method of the Optuna algorithm. The number of iterations was 50. Six important hyperparameters were selected for adjustment: max_depth, min_child_weight, subsample, num_leaves, learing_rate, and n_estimators. max_depth denotes the maximum depth of the tree; min_child_weight denotes the minimum leaf weight; subsample denotes the sampling rate of training samples, which can prevent the model from overfitting; n_estimators denotes the number of weak learners; num_leaves denotes the maximum number of leaves of the tree, which is one of the most important parameters to control the complexity of the model; and learing_rate denotes the learning rate. The other hyperparameters were kept as default values, and the results of the classifier hyperparameter search are shown in Table 8.

**Figure 9:**
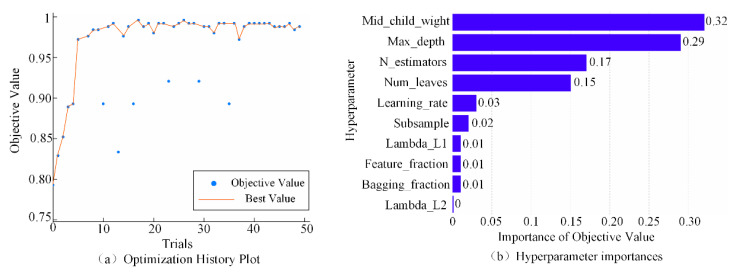
The Optuna dashboard. (**a**) The optimization history plot. (**b**) The importance of hyperparameters.

Optuna also provides a web dashboard for visualizing and analyzing the study, as shown in Figure 9. As can be seen in Figure 9a, the recognition accuracy exceeded 95% when the number of iterations reached about six and increased slowly in subsequent training. As can be seen in Figure 9b, min_child_weight and max_depth were the most important hyperparameters that affect the model performance.

**Figure 10:**
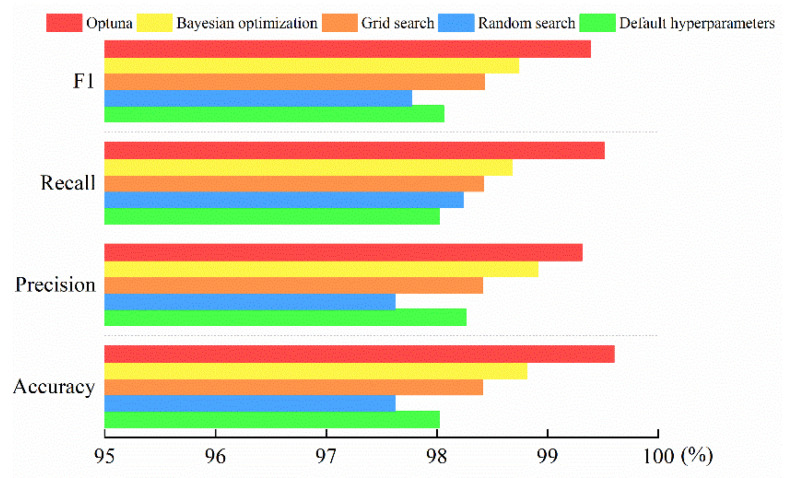
The experimental results of different hyperparameter optimization methods.

To verify the superiority of the Optuna optimization algorithm in determining hyperparameters, it was compared with default hyperparameters, random search algorithm, grid search algorithm, and the Bayesian optimization algorithm. The experimental results of different hyperparameter optimization methods are given in Figure 10.

From Figure 10, it can be seen that the hyperparameter optimization method based on the Optuna algorithm outperformed the other five hyperparameter optimization algorithms in all indexes. The new method effectively determined the optimal values of important hyperparameters in LightGBM and improved the accuracy of power fingerprint identification.

### 6.5. Comparison of the Impact of Entropy Features on Classification Performance

To verify the effectiveness of the entropy features proposed by the new method, a set of the 50-dimensional original feature set without entropy features and a set of the 56-dimensional original feature set with entropy features were set in this paper. After feature selection for these two sets of original feature sets, the optimal feature subset of 25 dimensions and the optimal feature subset of 23 dimensions were determined. These two sets of original feature sets and optimal feature subsets were input to the OPT–LightGBM classifier for identification. The experimental results are shown in Table 9.

As can be seen from Table 9, the new method uses more types of entropy features in the original feature set, wherein the optimal feature subset contains five entropy features. Compared with the original feature set and the optimal feature subset without entropy features, it improved the accuracy by 0.74% and 1.79%, respectively. The entropy feature can reflect the complexity and self-similarity of current signals of different electrical equipment in stable operation, which can better improve the identification accuracy of power fingerprints.

**Figure 11:**
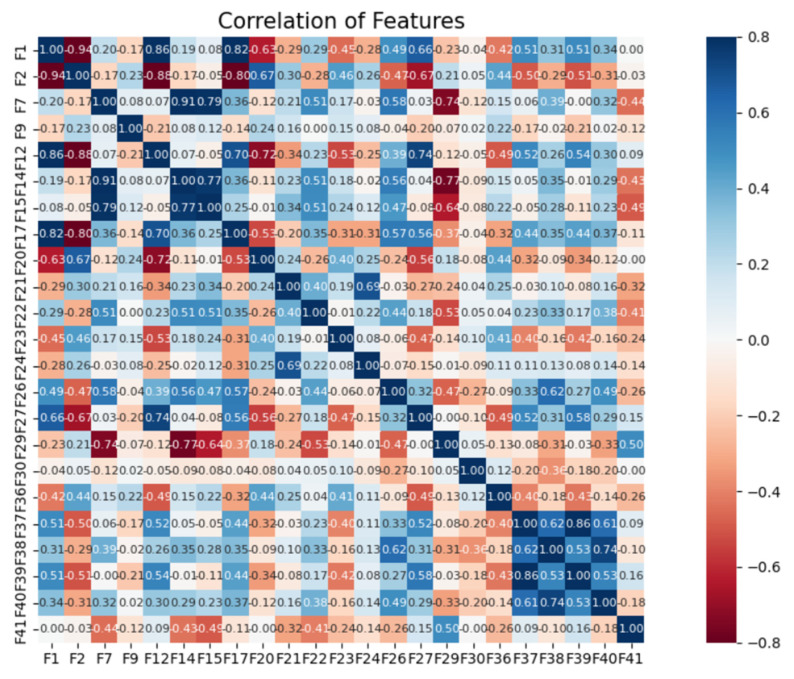
The correlation between entropy features in the heat map.

The correlation between entropy features is well reflected in the heat map shown in Figure 11. The scale on the right side of the heat map shows the shades of color corresponding to the different correlation coefficients, making it easy to see the correlation between features through the visual form. As can be seen from the graph, the correlation between the five entropy features takes a value between −0.2–0.69 compared to the other features, indicating that the correlation between the features is low, and therefore these five entropy features were retained when feature selection was carried out.

### 6.6. Comparison of the Performance of Each Classifier under an Imbalanced Dataset

As shown in Table 5, the COOLL dataset has the problem of class imbalance. To further verify the classification performance of the OPT–LightGBM model proposed in this paper on the imbalanced dataset, the method was compared with SVM, KNN, DT, RF, GBDT, XGBoost, and no-optimization LightGBM. The 23-dimensional optimal feature subset in the experiments was determined from the original 56-dimensional feature set after the same feature selection. Table 10 shows the experimental results of the classification performance of different classifiers.

**Figure 12:**
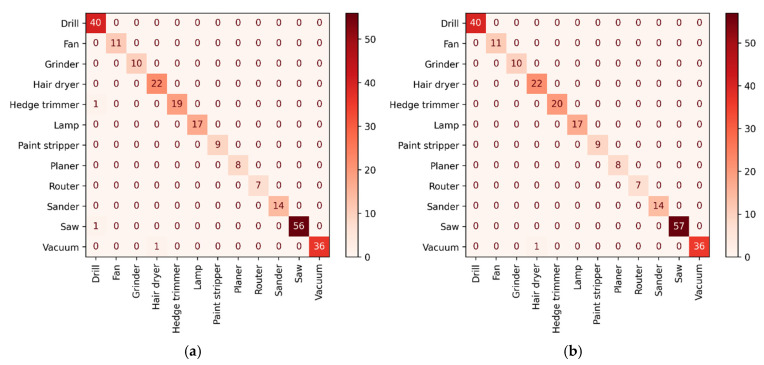
The confusion matrix for appliance loads of the COOLL dataset. (**a**) Confusion matrix based on the no-optimization LightGBM model. (**b**) Confusion matrix based on the OPT–LightGBM model.

As can be seen from Table 10, the classifiers such as SVM, KNN, and DT had lower metrics, while the OPT–LightGBM model, which considers the class imbalance problem, had a significant improvement in various classification performance metrics compared to RF, GBDT, XGBoost, and LightGBM. To further compare the differences between LightGBM and the proposed method, a confusion matrix of the experimental results is given in Figure 12.

As can be seen from Figure 12, LightGBM was confusing in the recognition process of the appliances Drills, Saw, and Hedge Trimmers. Compared with LightGBM, OPT–LightGBM was able to identify both appliances normally, and the overall recognition accuracy of electric fingerprint was 99.60%. The experimental results show that OPT–LightGBM had better classification accuracy than other classifiers on imbalanced datasets and effectively improved the generalization ability of the model.

### 6.7. Analysis and Discussion

To reduce the pressure of feature computation and network bandwidth of the power fingerprint identification system, an optimization method of LightGBM power fingerprint extraction and identification based on entropy features was studied.

From Table 6 and Table 7, it can be seen that compared to other feature selection algorithms, the modified Boruta algorithm reduced the original 56-dimensional feature set to a 23-dimensional optimal feature subset. The modified Boruta algorithm achieved 99.60% identification accuracy after feature selection, while the embedded feature selection method achieved 98.81% identification accuracy. Compared with this, the method in this paper improved the accuracy by 0.79%. In addition, it can be seen from Table 9 and Figure 11 that the correlation between the five entropy features retained after feature selection was low, which was good for improving the power fingerprint identification.

As can be seen in Figure 9a, Optuna was able to find the best hyperparameter configuration in a limited number of runs (only 50 iterations). LightGBM with Optuna optimization showed better power fingerprint identification compared to other optimization algorithms, as shown in Figure 10. As can be seen from Table 10, LightGBM was still able to maintain a high identification accuracy of 99.60% in the face of imbalanced power fingerprint data. Although the identification accuracies of GBDT, XGBoost, and LightGBM, which performed relatively well in the table, were 98.02%, 98.41%, and 98.81%, respectively, the method in this paper improved the accuracies by 1.58%, 1.19%, and 0.79%, respectively.

Among the 11 types of electrical devices in the COOLL dataset, different electrical devices will exhibit the same waveform between stable operations, resulting in similar electrical fingerprints, such as Drill, Saw, and Hedge Trimmer, as shown in Figure 3. However, this paper took into account the transient characteristics generated by electrical devices in transient states such as F26, F29, and F30, thus improving the differentiation between similar electrical devices. From Figure 12a,b, it can be seen that Drill, Saw, and Hedge Trimmer were 100% correctly classified in the identification results of OPT–LightGBM, which reduced the misclassification rate of similar appliances. The experimental results show that OPT–LightGBM can identify similar electrical devices well.

## 7. Conclusions

In this paper, we propose an optimized LightGBM power fingerprint extraction and identification method based on entropy features. The main advantages of the new method are as follows:

- A modified Boruta algorithm was used to feature select the original feature set containing six entropy features and to construct the optimal feature subset, which further improved the optimization-seeking efficiency of feature selection and reduced the impact of redundant features on the classification performance of the classifier. The experimental results showed that the five entropy features retained after feature selection significantly improved power fingerprint identification.
- The optimal feature subset replaced the original signal and the original feature set and was uploaded to the upper system, effectively reducing the amount of data transmission and feature computation required for edge devices and reducing the overall communication and hardware cost of the system.
- A lightweight power fingerprint identification model for class-imbalanced samples was constructed. The LightGBM loss function was improved, and its parameters were optimized using the Optuna optimization algorithm. The experimental results show that the method improved the accuracy of power fingerprint identification on imbalanced datasets and effectively verified the model’s generalization ability.

The experimental results show that the method reduced the pressure on the feature computation and network bandwidth of the power fingerprint identification system while still maintaining more than 98% of the power fingerprint recognition accuracy. The new method can effectively promote the applicability of power fingerprint recognition technology in the actual field. In addition, with the ever-changing types of actual electrical equipment in the home, there is a higher demand for the ability to identify complex equipment applications for model recognition. The identification effect of the method in this paper on the simultaneous operation of multi-state loads requires further improvement, and the generality of the identification model requires further study.

## Figures and Tables

**Table 1:** The calculation formulas and feature numbers of current features.

Features	Formula	Feature Number	Features	Formula	Feature Number
Maximum value	$F_{1}=\max(x(n))$	F1	Peak	$F_{10}=\max|x(n)|$	F10
Minimum value	$F_{2}=\min(x(n))$	F2	Peak to peak value	$F_{11}=F_{1}-F_{2}$	F11
Mean value	$F_{3}=\frac{1}{N}\sum_{n=1}^{N}x(n)$	F3	Absolute mean	$F_{12}=\frac{1}{N}\sum_{n=1}^{N}\left|x(n)\right|$	F12
Variance	$F_{4}=\frac{1}{N-1}\sum_{n=1}^{N}\left(x(n\right)-F_{3}{)}^{2}$	F4	Square root amplitude	$F_{13}={\left(\frac{1}{N}\sqrt{\sum_{n=1}^{N}\left|x(n)\right|}\right)}^{2}$	F13
Standard deviation	$F_{5}=\sqrt{\frac{1}{N}\sum_{n=1}^{N}\left(x(n\right)-F_{3}{)}^{2}}$	F5	Waveform index	$F_{14}=\frac{\max|x(n)|}{F_{6}}$	F14
Root mean square	$F_{6}=\sqrt{\frac{1}{N}\sum_{n=1}^{N}{\left(x(n)\right)}^{2}}$	F6	Peak index	$F_{15}=\frac{N\max|x(n)|}{\sum_{n=1}^{N}\left|x(n)\right|}$	F15
Cliffness	$F_{7}=\frac{\sum_{n=1}^{N}{\left(x(n)-F_{3}\right)}^{3}}{(N-1)F_{5}{}^{3}}$	F7	Pulse index	$F_{16}=\frac{\max|x(n)|}{F_{13}}$	F16
Skewness	$F_{8}=\frac{NF_{6}}{\sum_{n=1}^{N}\left|x(n)\right|}$	F8	Clearance index	$F_{17}=\frac{\max|x(n)|}{F_{6}}$	F17
Sum of maximum and minimum values	$F_{9}=F_{1}+F_{2}$	F9	Energy	$F_{18}=\sum_{n=1}^{N}\left|{\left(x(n)\right)}^{2}\right|$	F18

**Table 2:** The calculation formulas and feature numbers of entropy features.

Features	Formula	Feature Number	Features	Formula	Feature Number
Shannon entropy	$F_{19}=-\sum_{n=1}^{N}p_{n}\log p_{n}$	F19	Fuzzy entropy	$F_{22}=\ln\Psi^{m}(r)-\ln\Psi^{m+1}(r)$	F22
Renyi entropy	$F_{20}=\frac{1}{1-\alpha }\log\sum_{n=1}^{N}p_{n}{}^{\alpha }$	F20	Permutation entropy	$F_{23}=-\sum_{n=1}^{m!}p_{n}\log p_{n}$	F23
Approximate entropy	$F_{21}=\Phi^{m}(r)-\Phi^{m+1}(r)$	F21	Sample entropy	$F_{24}=-\ln\frac{B^{m+1}(r)}{B^{m}(r)}$	F24

**Table 3:** The feature categories and numbers of V-I trajectory features in different states.

Features	Feature Number	Feature Number
	Transient	Steady State
Current span	F25	F37
Area	F26	F38
Direction	F27	F39
Asymmetry	F28	F40
The curvature of the mean line	F29	F41
Self-intersection	F30	F42
The peak of the middle segment	F31	F43
The shape of the middle segment	F32	F44
Area of left and right segments	F33	F45
Variation of instantaneous admittance	F34	F46
The angle between the maximum point and the minimum point	F35	F47
The distance between the maximum point and the minimum point	F36	F48

**Table 4:** The feature categories and numbers of V-I trajectory features.

Features	Feature Number
The difference between the current span of the steady state and transient trajectory	F49
The difference between the area of the steady state and transient trajectory	F50
The difference between the asymmetry of the steady state and transient trajectory	F51
The difference between self-intersection of the steady state and transient trajectory	F52
The difference between the peak of the middle segment of the steady state and transient trajectory	F53
The difference between the area of the left and right segments of the steady state and transient trajectory	F54
The difference between the angle between the maximum point and the minimum point of the steady state and transient trajectory	F55
The difference between the distance between the maximum and minimum points of the steady state and transient trajectory	F56

**Table 5:** The COOLL dataset.

Appliance Labels	Appliances	Number of Appliances	Number of Signals
A	Drill	6	120
B	Fan	2	40
C	Grinder	2	40
D	Hair dryer	4	80
E	Hedge trimmer	3	60
F	Lamp	4	80
G	Paint stripper	1	20
H	Planer	1	20
I	Router	1	20
J	Sander	3	60
K	Saw	8	160
L	Vacuum	7	140

**Table 6:** Comparison of different feature selection algorithms.

Model	Optimal Feature Dimension	Accuracy (%)	Recall (%)	Precision (%)	F1-Score (%)
No feature selection	56	97.62	98.24	97.62	97.77
Correlation coefficient	30	96.83	96.83	97.72	97.03
REF	25	97.22	97.22	97.87	97.36
GA	28	96.43	96.43	97.57	96.70
Embedded (LightGBM)	23	98.81	98.90	98.90	98.82
Modified Boruta	23	99.60	99.77	99.64	99.70

**Table 7:** Comparison of the amount of data of different transmission types.

Transmission Data Type	Amount of Data Transmission for a Set of Feature Sets (Byte)
Original signal	2,744,650
Original feature set	1591
Optimal feature subset	1118

**Table 8:** The result of hyperparameter optimization.

Model	Optimized Hyperparameters	Default Hyperparameters
LightGBM	‘max_depth’: 7, ‘subsample’: 0.8, ‘num_leaves’: 22, ‘learning_rate’: 0.018, ‘min_child_wight’: 1.12, ‘n_estimators’: 146	‘lambda_l1’: 0.5, ‘lambda_l2’: 0.5, ‘bagging_fraction’: 1, ‘feature_fraction’: 1, ‘num_threads’: 2

**Table 9:** Comparison of experimental results between different feature sets.

Model	Original Feature Dimension	With or Without Entropy Features	Entropy Feature Type of Original Feature Set	Classification Accuracy (%)	Optimal Feature Dimension	Entropy Feature Number of Optimal Feature Set	Classification Accuracy (%)
OPT–LightGBM	50	With	0	96.88	25	0	97.81
	56	Without	6	97.62	23	5	99.60

**Table 10:** Comparison of experimental results of the classification performance of different classifiers.

Model	Feature Subset Dimension	Accuracy (%)	Recall (%)	Precision (%)	F1-Score (%)
SVM	23	94.44	96.97	96.14	96.47
KNN	23	95.63	95.95	95.88	95.82
DT	23	97.22	97.07	97.27	97.01
RF	23	98.01	97.68	98.81	98.19
GBDT	23	98.02	98.94	99.07	98.99
XGBoost	23	98.41	98.46	98.26	98.27
LightGBM	23	98.81	98.90	98.90	98.82
OPT–LightGBM	23	99.60	99.77	99.64	99.70

## Data Availability

No data was used for the research described in the article.

## Abbreviations

The following abbreviations are used in this manuscript:

LightGBM	Light gradient boosting machine
NILD	Non-intrusive load disaggregation
STFT	Short-time Fourier transform
WT	Wavelet transform
RFE	Recursive elimination
RF	Random forest
DT	Decision tree
SVM	Support vector machine
KNN	K-nearest neighbor
CNN	Convolutional neural network
RNN	Recurrent neural network
GBDT	Gradient boosting decision tree
XGBoost	Extreme gradient boosting
GA	Genetic algorithm

## Appendix A

The trajectory was divided into two parts named A and B according to the maximum and minimum points of voltage, respectively. vmax is the first point of the trajectory, and let the first point is the maximum point. NT refers to the number of data points.

(A1) $$A=\left\{\left(V_{q},I_{q}\right)|q\in 1,2,\ldots ,v\min\right\}$$

(A2) $$B=\left\{\left(V_{q},I_{q}\right)|q\in v\min+1,\cdots ,NT\right\}$$

**Table A1:** The calculation formulas and feature numbers of V-I trajectory features in Table 3.

Formula	Formula
$F_{25}=\max(I(s))-\min(I(s))$	$F_{37}=\max(I(z))-\min(I(z))$
$F_{26}=\sum_{i}\frac{1}{2}|V_{j}(s)-V_{i}(s)|\left(\left|I_{i^{\prime }}(s)-I_{i}(s)|+|I_{j^{\prime }}(s)-I_{j}(s)\right|\right)$	$F_{38}=\sum_{i}\frac{1}{2}|V_{j}(z)-V_{i}(z)|\left(\left|I_{i^{\prime }}(z)-I_{i}(z)|+|I_{j^{\prime }}(z)-I_{j}(z)\right|\right)$
$F_{27}=\sum_{s}\frac{1}{2}\left(V(s+1)-V(s)\right)\left(I(s+1)-I(s)\right)$	$F_{39}=\sum_{z}\frac{1}{2}\left(V(z+1)-V(z)\right)\left(I(z+1)-I(z)\right)$
$F_{28}=Hausdorff\;\mathrm{distance}\left\{\left[V(s),I(s)\right],(-1)\left[V(s),I(s)\right]\right\}$	$F_{40}=Hausdorff\;\mathrm{distance}\left\{\left[V(z),I(z)\right],(-1)\left[V(z),I(z)\right]\right\}$
$F_{29}=\{(X,Y)|X=\frac{1}{2}V_{i}(s)+V_{i^{\prime }}(s)),Y=\frac{1}{2}I_{i}(s)+I_{i^{\prime }}(s))\}$	$F_{41}=\{(X,Y)|X=\frac{1}{2}V_{i}(z)+V_{i^{\prime }}(z)),Y=\frac{1}{2}I_{i}(z)+I_{i^{\prime }}(z))\}$
$F_{30}=((i\vec{j})\times (i\vec{i^{\prime }}))\cdot ((i\vec{j})\times (i\vec{j^{\prime }}))$	$F_{42}=((i\vec{j})\times (i\vec{i^{\prime }}))\cdot ((i\vec{j})\times (i\vec{j^{\prime }}))$
$\begin{array}{l} F_{31}=\max(\underset{r}{\max}\left(I_{r}(s)-f_{A}[V_{r}(s)]\right),\underset{t}{\max}\left(I_{t}(s)-f_{B}[V_{t}(s)]\right)),\\ r\in (m_{a},\ldots ,m_{a}+n_{a}-1),t\in (m_{b},\ldots ,m_{b}+n_{b}-1) \end{array}$	$\begin{array}{l} F_{43}=\max(\underset{r}{\max}\left(I_{r}(z)-f_{A}[V_{r}(z)]\right),\underset{t}{\max}\left(I_{t}(z)-f_{B}[V_{t}(z)]\right)),\\ r\in (m_{a},\ldots ,m_{a}+n_{a}-1),t\in (m_{b},\ldots ,m_{b}+n_{b}-1) \end{array}$
$F_{32}=std(I_{r}(s))+std(I_{t}(s))$	$F_{44}=std(I_{r}(z))+std(I_{t}(z))$
$F_{33}=F_{26-\mathrm{left}}+F_{26-\mathrm{right}}$	$F_{45}=F_{38-\mathrm{left}}+F_{38-\mathrm{right}}$
$F_{34}=std(\frac{I(s)}{V(s)})$	$F_{46}=std(\frac{I(z)}{V(z)})$
$F_{35}=\sqrt{|\max(V(s))-\min(V(s)){|}^{2}+|\max(I(s))-\min(I(s)){|}^{2}}$	$F_{47}=\sqrt{|\max(V(z))-\min(V(z)){|}^{2}+|\max(I(z))-\min(I(z)){|}^{2}}$
$F_{36}=\arctan(\frac{\max(I(s))-\min(I(s))}{\max(V(s))-\min(V(s))})$	$F_{48}=\arctan(\frac{\max(I(z))-\min(I(z))}{\max(V(z))-\min(V(z))})$

I(s) and I(z) are the steady-state and transient current waveforms of the load, respectively, whereas $i^{\prime }$ and $j^{\prime }$ denote the points on part B for which the voltage is closest to the two consecutive points i and j, respectively. The average voltage and current on points i and $i^{\prime }$ are taken as X and Y, respectively, which refer to the coordinate of point i. $F_{30}$ and $F_{42}$ judge whether there is an intersection between i, $i^{\prime }$, j, and $j^{\prime }$ to determine the number of self-intersections in the steady state and transient state. f[x] represents a straight-line expression determined by two points; $m_{a}$ and $m_{b}$ are the first point position of the middle segment in parts A and B, respectively; and $n_{a}$ and $n_{b}$ refer to the number of data points in the middle segment of parts A and B, respectively. The left and right subscripts represent the left and right segments of the trajectory, respectively. std represents the standard deviation function.

**Table A2:** The calculation formulas and feature numbers of V-I trajectory features in Table 4.

Formula	Formula
$F_{49}=F_{25}-F_{37}$	$F_{53}=F_{31}-F_{43}$
$F_{50}=F_{26}-F_{38}$	$F_{54}=F_{33}-F_{45}$
$F_{51}=F_{30}-F_{42}$	$F_{55}=F_{35}-F_{47}$
$F_{52}=F_{29}-F_{41}$	$F_{56}=F_{36}-F_{48}$
